# Supplementary material for: Health systems impacts of the COVID-19 pandemic on malaria control program implementation and malaria burden in Benin: A mixed-method qualitative and mathematical modelling and study
Source: PLOS Glob Public Health. 2024 Feb 2;4(2):e0002197. doi: 10.1371/journal.pgph.0002197 (PMC10836704; doi:10.1371/journal.pgph.0002197)
Supplement: S1 Text — (DOCX) [file pgph.0002197.s001.docx]

**S1 Text**

Key Informant interview guide.

| **N°** | **QUESTION** | **RESPONSE** |
| --- | --- | --- |
|  | **ROLE** |  |
| 1 | What is your role in the fight against malaria in Benin in your current job? |  |
| 2 | What activities do you undertake as part of your work in the fight against malaria? |  |
| 3 | In terms of malaria control, how has your work been affected or changed by the Covid-19 pandemic?    Prompt: e.g., availability of resources to fulfill the role, new tasks, change in responsibilities. |  |
| 4 | Overall, from what you know, how has the current malaria program been affected by the Covid-19 pandemic?    Prompt: e.g., supply chain for malaria prevention and case management (nets, rapid diagnostic tests, antimalarial drugs), impact on human resources in the health sector, detour of resources from the health system to new priorities. |  |
| 5 | What steps have you taken to try to overcome some of the changes in your work? |  |
| 6 | Do you know other actions that have been undertaken elsewhere in Benin Republic to overcome the impacts on malaria control during the pandemic? |  |
| 7 | In your opinion, what are some of the enabling factors and some of the barriers to sustaining malaria control during the pandemic? |  |
| 8 | Is there anything else you would like to add regarding the pandemic and malaria control? |  |
| 9 | Do you have any questions for us? |  |
