# Supplementary material for: Health systems impacts of the COVID-19 pandemic on malaria control program implementation and malaria burden in Benin: A mixed-method qualitative and mathematical modelling and study
Source: PLOS Glob Public Health. 2024 Feb 2;4(2):e0002197. doi: 10.1371/journal.pgph.0002197 (PMC10836704; doi:10.1371/journal.pgph.0002197)
Supplement: S2 Text — (DOCX) [file pgph.0002197.s002.docx]

**S2 Text**

Quotes related to the impact of COVID-19 on malaria control in French, presented in order of inclusion in the main text.

1. *“Tout ceci pour libérer et minimiser la propagation de la maladie au sein du personnel au niveau de notre structure sanitaire, histoire de laisser poursuivre l’offre des soins et les consultations à soins curatives.”* (Cotonou, niveau national)
2. “*Quand on s’est déplacé vers les gens pour leur offrir les intrants, cela a été meilleure. La distribution va donc plus aux bénéficiaires que lorsqu’on remettait tout un lot pour toute une famille à une personne X, qui transporte pour aller faire une redistribution secondaire aux autres. Chaque famille recevait directement ses intrants, ce qui a évité le ralentissement.*” (Cotonou, niveau national)
3. “*Nous avons pris des mesures qu’il faut pour pouvoir traiter nos malades normalement. Si ce n’est pas la phobie et la panique de la population, ils devaient fréquenter les centres de santé. Mais, on dit dans la population que c’est dans les centres de santé qu’il y a la maladie. C’est cette « fausse » information même qui a fait que les gens ont fui les centres de santé.*” (Covè, niveau communautaire)
4. “*Au début, cette rupture ne dépend pas forcément de la Covid-19 directement à notre niveau peut-être au niveau national. Vu que les produits ne sont pas fabriqués ici au Bénin, c’est toujours importé.*” (Covè, niveau zone sanitaire)
5. “*Mais en matière de CTA, on était obligé d’aller vers la quinine pour les quelques traitements dans cette période avant l’approvisionnement de la zone en TDR et CTA.”* (Ouinhi, niveau communautaire)
