# Supplementary material for: Health systems impacts of the COVID-19 pandemic on malaria control program implementation and malaria burden in Benin: A mixed-method qualitative and mathematical modelling and study
Source: PLOS Glob Public Health. 2024 Feb 2;4(2):e0002197. doi: 10.1371/journal.pgph.0002197 (PMC10836704; doi:10.1371/journal.pgph.0002197)
Supplement: Attachment — Submitted filename: BeninCOVID_Response.docx [file pgph.0002197.s004.docx]

**Reply to reviewer comments**

Reviewer #1

The manuscript is well written and the finding support the conclusion.

I have some concerns that I am suggesting that may help authors to improve transparency.
Materials and methods are suggested to be reported in separate sections as per the STOBE guidelines.

*>> We thank the reviewer for this suggestion. We have reviewed the STROBE guidelines for cross-sectional studies (most aligned with our study design), as well as the Standards for Reporting Qualitative Research guidelines (SRQR, doi: 10.1097/ACM.0000000000000388) to verify that we adhered to those guidelines where applicable. We identified one area of shortfall, the number of key informants interviewed for our study, which has now been added (L158-159 and Table 1).*

The same goes for the results to slip into the separate sub-sections that support answering the research questions.
*>> The Results section is currently structured as the reviewer suggested: sections 1 and 2 (description of the health system and results from KIIs) correspond to Obj 1, and section 3 (model results) correspond to Obj 2.*

The author may find this paper interesting to cite as the possible impact of covid on mosquito-born disease is discussed in this article: <https://doi.org/10.18332/popmed/145535>

the impact of the covid on the health system and the nurses in the context of a developing country are evident in the following articles and the author may find them interesting to cite:
<https://doi.org/10.1186/s43045-021-00103-x>
<https://doi.org/10.1016/j.heliyon.2023.e13162>
<https://doi.org/10.1111/inr.12802>
<https://doi.org/10.1371/journal.pone.0274965>

*>> We thank the reviewer for suggesting these interesting papers on the impact of COVID-19 on mosquito-borne diseases in Bangladesh and on the mental health of nurses in Bangladesh, however, we have decided to not discuss them in our manuscript to maintain focus on the sub-Saharan African context.*

Reviewer #2

Appreciate the authors for this study to find out the impact of Covid19 on malaria control programs by using mixed methods in three different levels of health system. The authors have presented the expected and unexpected and also positive negative impacts at the various levels of health system and formulated an agent-based model of malaria epidemiology to assess the impacts of treatment disruption on malaria. The effort put in by he authors are laudable.

*>> We thank the reviewer for their kind words and are please that they concur with the goals of this study.*

The limitation is that this study was done in the early stage of covid when the health system was unaware of many aspects also about resilience. Later on, lot of developments have happened regarding the disease Covid 19, the mitigation strategies, how to bring in resilience etc. So, this manuscript may appear as if not relevant for today’s situation. But this study could be an inspiration to have a further look at the same thing at a later date thereby understanding more about the impact of Covid 19 on Malaria control programs in a changed health system and the lesson could have far reaching importance like prevention of further pandemics. So authors can elaborate more on the limitations of the study and the scope for further studies related to this one.

*>> We thank the reviewer for pointing this out. We agree that our study does not apply to the situation in current-day Benin and follow-up studies on the long-term impacts of COVID-19 on the health system and malaria control programs would be invaluable. These points have been added to the manuscript on L478-493.*

In addition more details to be given related to the integration of qualitative and quantitative methods in the context.

*>> More details on how the qualitative interviews and mathematical model complement each other has been added to the Introduction (L114-116) and Methods (L219-220).*
